# Liverpool School of Tropical Medicine

**Published:** 1907-08-24

**Authors:** 


### Tropical Diseases.

### LIVERPOOL SCHOOL OF TROPICAL MEDICINE.

A new expedition has been fitted out by the Liverpool School of Tropical Medicine to proceed to the West Coast of Africa and investigate black-water fever. It consists of Drs. Yorke and Barrett, and they were recently entertained at a farewell dinner given at Liverpool. The disease is one of great improtance to many of our Colonies in Africa, and as its etiology is somewhat obscure it is to be hoped that the investigators will reach some definite conclusions. In addition to this expedition, two others from the same school are at present abroad, one in Peru studying yellow fever, the other in Africa studying sleeping sickness.

